# Risk of SARS-CoV-2 exposure among hospital healthcare workers in relation to patient contact and type of care

**DOI:** 10.1177/14034948211022434

**Published:** 2021-06-19

**Authors:** Susanna Klevebro, Fuad Bahram, K. Miriam Elfström, Ulrika Hellberg, Sophia Hober, Simon Kebede Merid, Inger Kull, Peter Nilsson, Per Tornvall, Gang Wang, Kalle Conneryd Lundgren, Sari Ponzer, Joakim Dillner, Erik Melén

**Affiliations:** 1 Department of Clinical Science and Education Södersjukhuset, Karolinska Institutet, Stockholm, Sweden; 2 Sachs’ Children and Youth Hospital, Stockholm, Sweden; 3 Clinical Reseach Center, Södersjukhuset, Sweden; 4 Karolinska University Hospital, Sweden; 5 Department of Protein Science, KTH Royal Institute of Technology, SciLifeLab, Stockholm, Sweden; 6 Department of Integrated Traditional Chinese and Western Medicine, West China Hospital, Sichuan University, Sichuan, China

**Keywords:** Antibodies, COVID-19, healthcare workers, health personnel, SARS-CoV-2, seroepidemiologic studies, seroprevalence

## Abstract

**Aim:**

We aimed to assess prevalence of IgG antibodies to severe acute respiratory syndrome coronavirus-2 (SARS-CoV-2) and factors associated with seropositivity in a large cohort of healthcare workers (HCWs).

**Methods:**

From 11 May until 11 June 2020, 3981 HCWs at a large Swedish emergency care hospital provided serum samples and questionnaire data. Presence of IgG antibodies to SARS-CoV-2 was measured as an indicator of SARS-CoV-2 exposure.

**Results:**

The total seroprevalence was 18% and increased during the study period. Among the seropositive HCWs, 11% had been entirely asymptomatic. Participants who worked with COVID-19 patients had higher odds for seropositivity: adjusted odds ratio 1.96 (95% confidence intervals 1.59–2.42). HCWs from three of the departments managing COVID-19 patients had significantly higher seroprevalences, whereas the prevalence among HCWs from the intensive care unit (also managing COVID-19 patients) was significantly lower.

**Conclusions:**

**HCWs in contact with SARS-CoV-2 infected patients had a variable, but on average higher, likelihood for SARS-CoV-2 infections.**

## Background

On 27 February 2020 the first confirmed case of infection with severe acute respiratory syndrome coronavirus-2 (SARS-CoV-2) in Stockholm, Sweden was diagnosed [1]. The number of patients in need of hospital care due to the disease caused by SARS-CoV-2, Coronavirus Disease 2019 (COVID-19), in the Stockholm region escalated in March and the beginning of April and started to decline in May 2020 [2]. Healthcare workers (HCWs) with direct patient contact are more exposed to the virus and thereby have a higher risk of contracting SARS-CoV-2 compared to the general population [3–5]. Presence of SARS-CoV-2 IgG antibodies indicates a previous SARS-CoV-2 infection. A global review of previous studies published up to August 2020 found an overall seroprevalence of SARS-CoV-2 antibodies among HCWs to be 8.7% (95% confidence intervals (CI): 6.7– 0.9%) [6]. The aim of this study was to assess the seroprevalence in a large cohort of HCWs at a Swedish emergency care hospital after the first wave of COVID-19 patient admissions. The secondary aim was to investigate factors associated with seropositivity.

## Methods

### Design and setting

This cross-sectional study was initiated as a part of a regional study of HCWs conducted in the major emergency care hospitals in Stockholm. Stockholm South General Hospital (Södersjukhuset) has one of the largest emergency care departments in Northern Europe with about 550 in-patient beds. The hospital provides both general and specialized acute as well elective care. From 27 February 2020, when the first case was verified in the region, until 30 June 2020, 1384 patients with confirmed SARS-CoV-2 infection had been admitted to the hospital (see Supplemental Figure). Guidelines recommended the use of face shields, face masks and plastic aprons during direct contact with patients suspected to be infected. Aerosol filtering face masks were recommended during aerosol generating procedures, and in the intensive care unit (ICU). HCWs who experienced any symptoms of infection were advised to stay at home until feeling well for at least two days but were not routinely tested for SARS-CoV-2 during this period. HCWs who had an ongoing SARS-CoV-2 infection confirmed with a PCR test were to follow the same guideline and stay at home at least one week following onset of symptoms.

### Participants and data collection

All employees working at Stockholm South General Hospital were invited to participate in this study and are referred to as HCWs in this study regardless of workplace or tasks at the hospital. Blood samples were collected at the hospital from 11 May to 11 June 2020. Self-reported information regarding current workplace, active work with infected patients (suspected or confirmed COVID-19 cases) and self-suspicion of prior SARS-CoV-2 infection were collected on questionnaires at the time of sampling.

Serum samples were prepared from whole blood, inactivated at 56°C for 30 min and stored at −20°C until analysis. IgG reactivity was measured towards three virus protein variants: spike trimers comprising the prefusion-stabilized spike glycoprotein ectodomain [7], spike S1 domain and nucleocapsid protein using multiplex bead array. Evaluation of the serology assay demonstrated a 99.2% sensitivity and 99.8% specificity [3]. The evaluation of the serology assay was based on 442 negative samples (defined as samples collected in the same region 2019 or earlier) and 243 samples from COVID-19 convalescents (defined as PCR-positive individuals sampled more than 16 days after a positive PCR test). The 442 negative samples included 26 individuals with confirmed infections of coronaviruses other than SARS-CoV-2.

### Statistical methods

Results are presented as prevalence. Association between a seropositive test and age (categorized as < 40 years and ⩾ 40 years), sex (female/ male), date of test (categorized as first, second and third to fifth week of the study) and active work with COVID-19 patients (categorized as no, yes and do not know) was examined in multivariable logistic regression model and presented as odds ratio (OR) with 95% CIs. To analyse if workplace was associated with seroprevalence each department was related to all other hospital departments in separate regression models adjusted for age (continuous), sex (female/male), date of test (first/second/third to fifth week) and active work with COVID-19 patients (no/yes/do not know). Statistical analyses were performed in R [8] using the glm function to run the regressions. The study was approved by the Stockholm Ethical Review Board (dnr 2020-0162 and 2020-02724). All participants provided written informed consent and serology testing results were conveyed to participants.

## Results

In total, 3981 HCWs participated in the study. Stockholm South General Hospital had 4641 employees in May 2020 and an estimate of the participation rate in this study is therefore 3981/4641 (86%). Of all participants, 0.7% (*n* = 26) had missing data in one or several of the variables collected on the questionnaire. Participants had a mean age of 45 years, 81% were female, and 64% responded that they had worked with patients with confirmed or suspected COVID-19 (Table I).

**Table I.** Characteristics of the study population.

Variables	Mean (SD)		
Age, *n* = 3981	46 (12)		
	*n* (%)	*n* (%)	*n* (%)
	Female	Male	
Sex, *n* = 3981	3210 (81)	771 (19)	
	Yes	No	Do not know
Active work with COVID-19 patients, *n* = 3965	2560 (65)	1122 (28)	283 (7)
Self-suspicion of prior SARS-CoV-2 infection, *n* = 3977	1042 (26)	1336 (34)	1599 (40)

**Figure 1.**
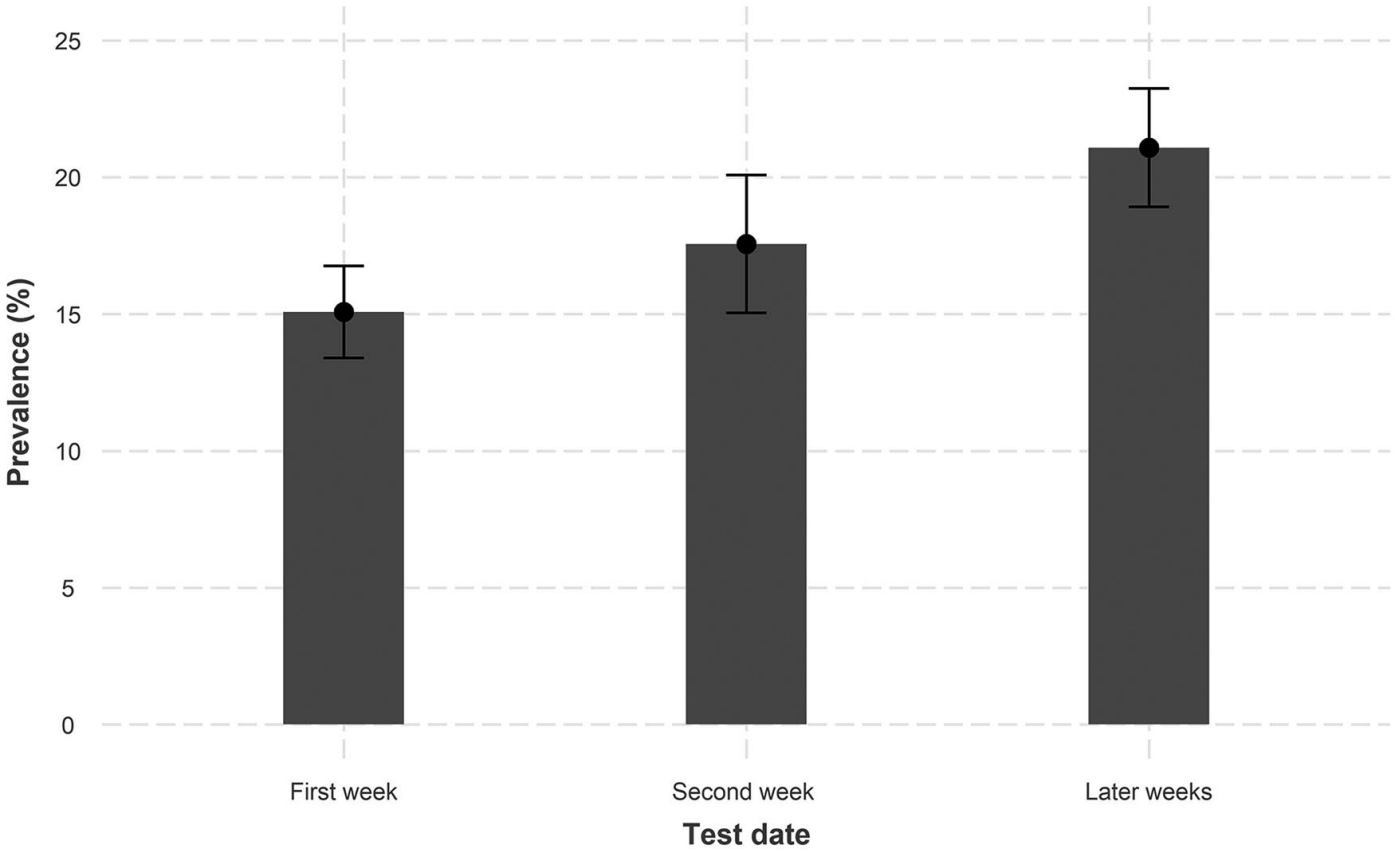
Prevalence of SARS-CoV-2 IgG antibodies by test date. Divided into first week, *n* = 1738; second week, *n* = 877; later weeks (week 3–5), *n* = 1366. Error bars shows 95% confidence intervals.

Among the participating HCWs, 704 (18%) were seropositive for IgG antibodies against SARS-CoV-2. The prevalence increased during the test period (Figure 1) and was 20% among men and 17% among women. Among HCWs below the age of 40 years the prevalence was 20%, compared to 16% in HCWs 40 years of age and above. Sex and age were not associated with the odds of a positive test (Table II). HCWs who reported that they had worked with COVID-19 patients had a higher prevalence of antibodies, 21% compared to 12% without exposure, adjusted odds ratio 1.96 (95% CI 1.59–2.42) (Table II).

**Table II.** Multivariable logistic regression presenting adjusted odds ratios (ORs) for a positive SARS-CoV-2 IgG test.

Variables	OR	95% CI	*p*-value
Age, higher or equal than 40 years^a^, *n* = 2506	1.10	0.92–1.30	0.29
Sex, male^b^, *n* = 771	1.12	0.91–1.37	0.28
Test date, second week^c^, *n* = 877	1.16	0.93–1.44	0.19
Test date, third to fifth week^c^, *n* = 1366	1.49	1.24–1.80	< 0.01
Active work with COVID-19 patients, yes^d^, *n* = 2560	1.96	1.59–2.42	< 0.01
Active work with COVID-19 patients, do not know^d^, *n* = 283	1.03	0.69–1.55	0.88

a Reference group: age < 40 years, *n* = 1475.

b Reference group: female, *n* = 3210.

c Reference group: test date, first week, *n* = 1738.

d Reference group: active work with COVID-19 patients, no, *n* = 1122.

CI, confidence interval.

**Figure 2.**
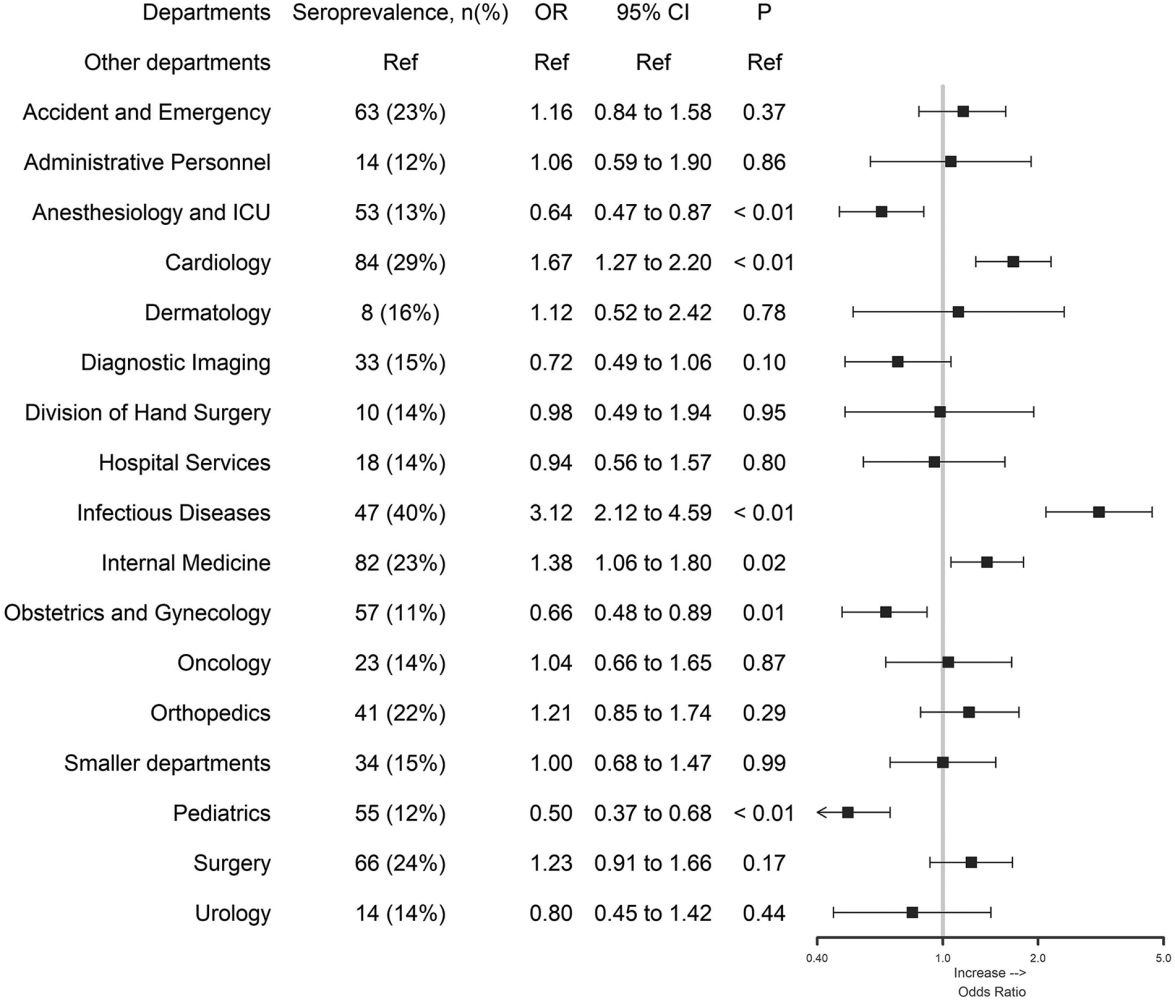
Differences between departments. The seroprevalence within the department is presented in parentheses, and the figure demonstrates odds ratio (OR) and 95% confidence intervals (CI) from multivariable regression models comparing each department with all other hospital departments as reference adjusted for sex (categorical), age (continuous), date of test (three categories: first week, second week, third to fifth week), active work with COVID-19 patients (three categories: no, yes, do not know). ‘Smaller departments’ include participants from smaller departments that do not belong in any of the listed departments.

Seroprevalence differed between departments: HCWs within the departments of Cardiology, Infectious Diseases and Internal Medicine had higher odds of being seropositive compared to HCWs at the rest of the hospital after adjustment for sex, age, date of test and active work with COVID-19 patients (Figure 2). In contrast, HCWs from the departments of Anaesthesiology/ICU, Obstetrics/Gynaecology and Paediatrics had lower odds of being seropositive compared to the rest of the hospital.

Self-reported suspicion of COVID-19 symptoms prior to the study was associated with higher prevalence of a positive test, 46% compared to 5.5% among HCWs who had not suspected previous infection. Among the seropositive HCWs, 11% had been asymptomatic (no suspicion of a previous SARS-CoV-2 infection).

## Discussion

This study found a prevalence of 18% of IgG antibodies against SARS-CoV-2 among HCWs in a large cohort of HCWs from Stockholm, Sweden sampled during the later phases of the first wave of the epidemic. The seroprevalence was similar to the 19% reported among 2149 HCWs at Danderyd Hospital, a hospital of equivalent size in the Stockholm area [3], whereas Karolinska University Hospital demonstrated a lower seroprevalence of 12% among 12,928 HCWs [9]. Globally, seroprevalences among HCWs have ranged from 0 to 45.3% [6]. Residents in Stockholm had a seroprevalence of around 10% during the time of our study [10]. Our results indicate that HCWs caring for COVID-19 patients had an increased odds of infection with SARS-CoV-2 during the first wave of the pandemic, but that this risk varied greatly between participants who had worked with COVID-19 patients and not, as well as between departments.

Self-reported high suspicion of infection has previously been associated with seropositivity [11–13]. In our study, 11% of the seropositive HCWs did not suspect a previous SARS-CoV-2 infection and were thus likely to have had an asymptomatic infection. The proportion of infected individuals that have asymptomatic infections is somewhat variable in the literature, with a recent systematic review estimating about 30% [14].

As in previous studies [3–5], an increased prevalence was seen in HCWs who had been in contact with COVID-19 patients. Lower prevalence in the Paediatrics and Obstetrics/Gynaecology departments was to be expected due to low exposure to patients infected with SARS-CoV-2 in these settings. HCWs from the departments of Cardiology, Infectious Diseases and Internal Medicine were highly involved in the care of COVID-19 patients. The higher odds for a seropositive test in these departments indicates an increased risk of infection for HCWs in these settings, which could not be explained by age, sex, reported work with COVID-19 patients or date of test. Possibly the use of personal protective equipment differed in these settings in the early stages of the pandemic. Other speculations are that HCWs in these departments were exposed to patients with diffuse symptoms in the early highly infectious stages of disease, and that the risk of transmission between co-workers therefore differed.

Interestingly, as in the study by Grant et al. [4], HCWs at Anaesthesiology/ICU had a lower seroprevalence and lower odds of being seropositive compared to the rest of the hospital adjusted for active work with COVID-19 patients. This could reflect the use of enhanced personal protective equipment or that the COVID-19 patients at the ICU may be in a later stage of the disease when viral loads of patients might have decreased.

During the spring, many HCWs were relocated within the hospital. Current workplace was self-reported, and a limitation of this study is that we were not able to fully distinguish primary (regular) workplace from workplace during the period of interest. Furthermore, we did not collect information regarding use of protective equipment or other potential exposures such as contact with infected persons outside work or with infected HCWs at work. The participation rate was high, but we do not have information enabling a description of non-participants. It is likely to believe that employees on parental leave and long-term sick leave to a high degree did not participate in the study since the sample collection was performed at the hospital. Comorbidities have been related to severe COVID-19 disease [15]. In this study we did not collect information regarding comorbidities and are not able to assess potential association with SARS-CoV-2 infection.

The number of COVID-19 patients admitted to our hospital peaked in the weeks before this study. The fact that the seroprevalences increased with calendar time suggests that the first wave of the epidemic was still ongoing during the time of this study. However, PCR testing for the virus was not performed.

## Conclusion

The prevalence of SARS-Cov-2 IgG antibodies was higher in HCWs caring for COVID-19 patients. However, the risk was highly variable between departments suggesting that other factors are important for the risk of the infection.

## Supplemental Material

sj-tif-1-sjp-10.1177_14034948211022434 – Supplemental material for Risk of SARS-CoV-2 exposure among hospital healthcare workers in relation to patient contact and type of care Click here for additional data file. Supplemental material, sj-tif-1-sjp-10.1177_14034948211022434 for Risk of SARS-CoV-2 exposure among hospital healthcare workers in relation to patient contact and type of care by Susanna Klevebro, Fuad Bahram, K. Miriam Elfström, Ulrika Hellberg, Sophia Hober, Simon Kebede Merid, Inger Kull, Peter Nilsson, Per Tornvall, Gang Wang, Kalle Conneryd Lundgren, Sari Ponzer, Joakim Dillner and Erik Melén in Scandinavian Journal of Public Health
