# Supplementary figures and images for: Risk of SARS-CoV-2 exposure among hospital healthcare workers in relation to patient contact and type of care
Source: Scand J Public Health. 2021 Jun 19;49(7):707–12. doi: 10.1177/14034948211022434 (PMC8521364; doi:10.1177/14034948211022434)

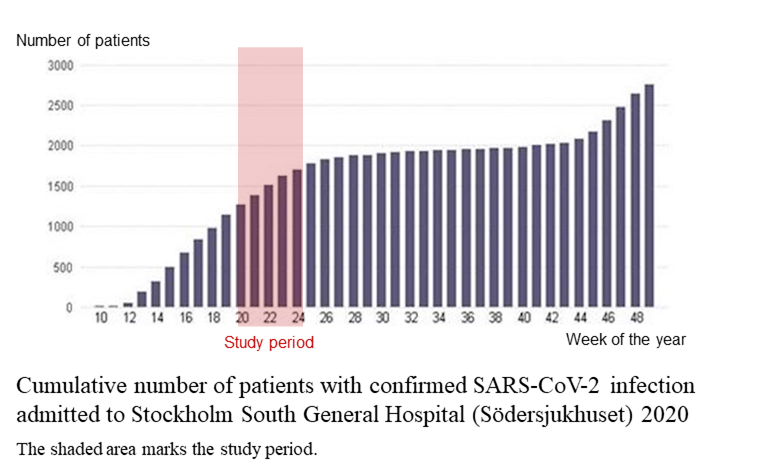

Supplement: sj-tif-1-sjp-10.1177_14034948211022434 – Supplemental material for Risk of SARS-CoV-2 exposure among hospital healthcare workers in relation to patient contact and type of care [file sj-tif-1-sjp-10.1177_14034948211022434.tif]
